# Paper: violence, abuse and exploitation among trafficked women and girls: a mixed-methods study in Nigeria and Uganda

**DOI:** 10.1186/s12889-022-13021-2

**Published:** 2022-04-20

**Authors:** Ligia Kiss, David Fotheringhame, Nambusi Kyegombe, Alys McAlpine, Ludmila Abilio, Agnes Kyamulabi, Eddy J. Walakira, Karen Devries, Clare Tanton

**Affiliations:** 1 Institute for Global Health, University College London, London, UK; 2 Independent Researcher, London, UK; 3 Global Health Department, London School of Hygiene and Tropical Medicine, London, UK; 4 Federal University of Campinas, Campinas, Brazil; 5 Makerere University Kampala, Kampala, Uganda

**Keywords:** Human trafficking, Modern-slavery, Violence, Migration, Africa, Adolescents, Mixed-methods

## Abstract

**Background:**

Africa is the global region where modern-slavery is most prevalent, especially among women and girls. Despite the severe health consequences of human trafficking, evidence on the risks and experiences of trafficked adolescents and young women is scarce for the region. This paper addresses this gap by exploring the intersections between violence, migration and exploitation among girls and young women identified as trafficking survivors in Nigeria and Uganda.

**Methods:**

We conducted secondary analysis of the largest routine dataset on human trafficking survivors. We used descriptive statistics to report the experiences of female survivors younger than 25 years-old from Nigeria and Uganda. We also conducted 16 semi-structured interviews with adolescents identified as trafficked in both countries. We used thematic analysis to explore participants’ perceptions and experiences before, during and after the trafficking situation.

**Results:**

Young female survivors of human trafficking in Nigeria and Uganda are exposed to a range of experiences of violence before migration, during transit and at destination. The qualitative data revealed that children and adolescents migrated to escape family poverty, violence and neglect. They had very low levels of education and most had their studies interrupted before migrating. Family members and close social contacts were the most common intermediaries for their migration. During transit, sexual violence and hunger were common, especially among Nigerians. Participants in both the quantitative and qualitative studies reported high levels of violence, deception, coercion, withheld wages and poor working conditions at destination. The adolescents interviewed in the qualitative study reported severe mental suffering, including suicide attempts. Only one reported the prosecution of perpetrators.

**Conclusions:**

Our findings suggest that interventions to prevent or mitigate the negative impact of adverse childhood experiences can contribute to preventing the trafficking of adolescents in Nigeria and Uganda. These interventions include social protection mechanisms, universal access to education, social service referrals and education of parents and carers. Importantly, effective prevention also needs to address the systemic conditions that makes trafficking of female adolescents invisible, profitable and inconsequential for perpetrators.

## Background

### Trafficking of female adolescents and youth in Africa

The International Labour Organisation (ILO) estimates that Africa is the world region where modern-slavery, including human trafficking, is most prevalent (7.6 per 1000 people), with forced marriages and sexual exploitation disproportionally affecting women and girls [1]. Human trafficking and violence have severe long-lasting consequences for the health and development of children and young people, and represent a burden to the future development of Low and Middle Income Countries (LMICs).

Although the term ‘modern slavery’ is not legally defined, it is increasingly used as an umbrella term to describe extreme labour exploitation, alternately referred to as forced labour and human trafficking. The most widely accepted definition, by the United Nations, describes human trafficking as the use of force, deception or coercion for the purposes of exploitation [2]. Neither “deception” or “exploitation” are, however, adequately defined in the Protocol. As a result, existing identification criteria and measurements often fail to grasp the complexity of the exploitative environment and concrete situations of workers [3].

In spite of this definitional fuzziness, evidence on human trafficking consistently shows its association with high levels of violence [4, 5]. Findings from a systematic review indicate that the prevalence of sexual violence ranges from 33 to 90% among survivors of sexual exploitation receiving post-trafficking assistance [4]. A study in the Mekong subregion estimated the prevalence of physical violence among adult female survivors receiving assistance at 41% [6]. One third of the participants in this study under the age of 18 experienced physical and/or sexual violence [7].

Violence is used by labour intermediaries and employers or traffickers to coerce or subjugate people into work [8]. The combination of high levels of violence with hazardous and intensive work means that few people come out of trafficking without long-term physical and psychological consquences [4, 9]. Trafficking in children and adolescents for labour exploitation is associated with both immediate, detectable damage, such as injuries, infections and illness, and serious long-term harm that often goes less noticed, including psychological disorders and impaired cognitive development [10, 11]. The mental health consequences of human trafficking are pervasive, and include high levels of PTSD, depression, anxiety and suicidal behaviour [4, 7, 11, 12].

Human trafficking often occurs in the context of economic migration, particularly low-wage migration. Exploitation, violence and abuse against low wage migrants, and especially among children, is associated with their limited bargaining power during transit and restricted work choices at destination. These risks are driven by the inequalities of economic development across countries and regions, alongside deeply embedded power imbalances between migrant workers and the agents and mechanisms that promote migration [9].

This paper explores the intersections between violence, migration and exploitation among girls and young women identified as trafficking survivors in Nigeria and Uganda. The results aim to inform human trafficking prevention and protection efforts that go beyond strategies that are widely used, albeit not supported by current evidence, such as pre-departure awareness raising [9, 13, 14].

## Research context

### Nigeria

The often disputed Global Slavery Index estimates [15] that almost 1.4 million Nigerians live in modern-slavery worldwide, with an estimated prevalence of 7.7 victims per 1,000 population (ref.). These estimates suggest that Nigeria, alongside the Democratic Republic of Congo, has the highest absolute number of modern-slaves in Africa accounting for more than a quarter of victims in the region [16]. The International Organization for Migration (IOM) also estimates that approximately 80% of girls arriving in Europe from Nigeria are potential victims of trafficking [17]. The Edo State is considered to be the main source region for human trafficking in Nigeria [18]. In the last two decades, there has also been a growth in internal trafficking from rural communities to cities such as Lagos, Abeokuta, Ibadan, Kano, Kaduna, Calabar and Port Harcourt [19]. Women and girls are trafficked mainly for domestic work and sexual exploitation. Reports indicate that government officials and security forces are often involved in cases of sexual exploitation and sex trafficking [20].

### Uganda

The Global Slavery Index estimates that the prevalence of trafficking in Uganda is 7.6 per 1000 population, a similar rate to the regional prevalence, which places the country as the 16th highest rate in Africa. Trafficking in children is a major concern in Uganda with vulnerable children at higher risk of being trafficked. These vulnerable children include orphaned children, children from poor households, children out of school, children who live or work on the street, children who are separated from their parents, children with low education, and those living in violent households [21].

Children as young as seven have been exploited through forced labour in a number of contexts and industries including agriculture, street vending, begging, bars and restaurants, and domestic service. Both boys and girls have also been exploited for commercial sex. Young women are also known to be the most vulnerable to transnational trafficking, usually through seeking employment as domestic workers in the Middle East and Asia. Young Ugandan women are also often exploited for forced sex work [22].

## Methods

### Data sources and collection procedures

We used mixed-methods data to examine the intersection of violence, migration and exploitation among trafficked adolescents in Nigeria and Uganda.

We analysed the International Organisation for Migration’s (IOM) Counter Trafficking Data Collaborative Global Data Hub on Human Trafficking (CTDC), previously named Trafficked Migrants Assistance Datasets (TMAD) [23]. This database contains routine data from cases of human trafficking assisted by IOM and its partner organisations across more than 164 destination countries. The data is collected by IOM staff and their partners through screening and assistance questionnaires, after the trafficking survivors have been referred to IOM [24]. We used these data to describe the type of exploitation, violence and abuse that girls and young women, who received post-trafficking assistance by IOM and their partners, experienced during migration. Data was available for 146 Nigerian and 95 Ugandan female adolescents and youth.

We analysed IOM data on Nigerian and Ugandan children (under 10 years old), adolescent girls (between 11 and 18 years-old) and young women (youth between 19 and 24 years-old) identified as trafficked by IOM and its partners. Field work was conducted between 2018 and 2019.

We also conducted semi-structured interviews with adolescent girls and key informants in Uganda and Nigeria to explore qualitatively the circumstances in which they entered trafficking, their migration journeys and their experiences at destination.

In Uganda, we interviewed eight girls who were under 18 when they migrated and were receiving post-trafficking assistance services in Kampala. Two of these girls were Rwandan and six were Ugandan. They were sampled from NGOs that provide post-trafficking assistance to trafficking survivors. The usual referral pathway to these services is through the police, who contact the NGO once they identify a person as a victim of human trafficking. The services provided by these NGOs include accommodation, basic needs, vocational skills training, medical services, psychological and spiritual counselling.

In Nigeria, we interviewed eight Nigerian girls who were under 18 when they migrated and were receiving post-trafficking assistance in services in the Northeast, North-central and Southwestern parts of the country.

Participants were identified by the post-trafficking assistance service’s staff and invited to participate. They were informed about the study and the sensitive nature of some of the questions. They were assured that they had the option of declining to participate and, if they agreed to participate, they were told they could delay or terminate the interview at any time. They were also informed that declining to participate would not have any effect on service provision. Interviewers were nationals from each country working in the local IOM offices. Members of the core team at LSHTM (AM and NK) delivered in person training on ethics and interview techniques to the IOM interviewers.

Interviews were conducted in private by IOM staff trained in qualitative interview techniques by the LSHTM team. No names or identifying information were included on any interview-related documents. All information relating to specific cases from any qualitative work was altered sufficiently to protect individuals’ identity. Participants were asked for their permission to use anonymous quotes in published materials. Names of places or persons that could potentially be used to identify participants were replaced by neutral terms and placed between brackets in all direct interview quotes reproduced in this article.

### Data analysis

We present descriptive statistics of the CTDC data. Due to the nature of these data (routine data for programmatic purposes), the dataset has a high proportion of missing values. We excluded missing values from the denominator to calculate the frequencies shown in the Results section. However, the denominators are included in the tables to facilitate interpretation. Despite the limitations of these data, we opted to include the quantitative results because of the unique nature of the dataset, which is the largest source of global data on trafficked people. Data from survivors is collected by IOM and their partners during screening and assistance interviews. They ask survivors questions about their experiences during trafficking, including their relationship with the exploiter defined as the agents who abused the vulnerability of a migrant for the purpose of exploiting him or her. Recruiters are defined as the intermediaries who facilitate migration and access to exploitative work. Receivers are the agents who facilitate migrants’ access to exploitative work at destination [25].

We applied thematic analysis to the qualitative data to explore the role of violence in migration decisions and the circumstances of exiting trafficking among adolescents.

**Fig. 1:**
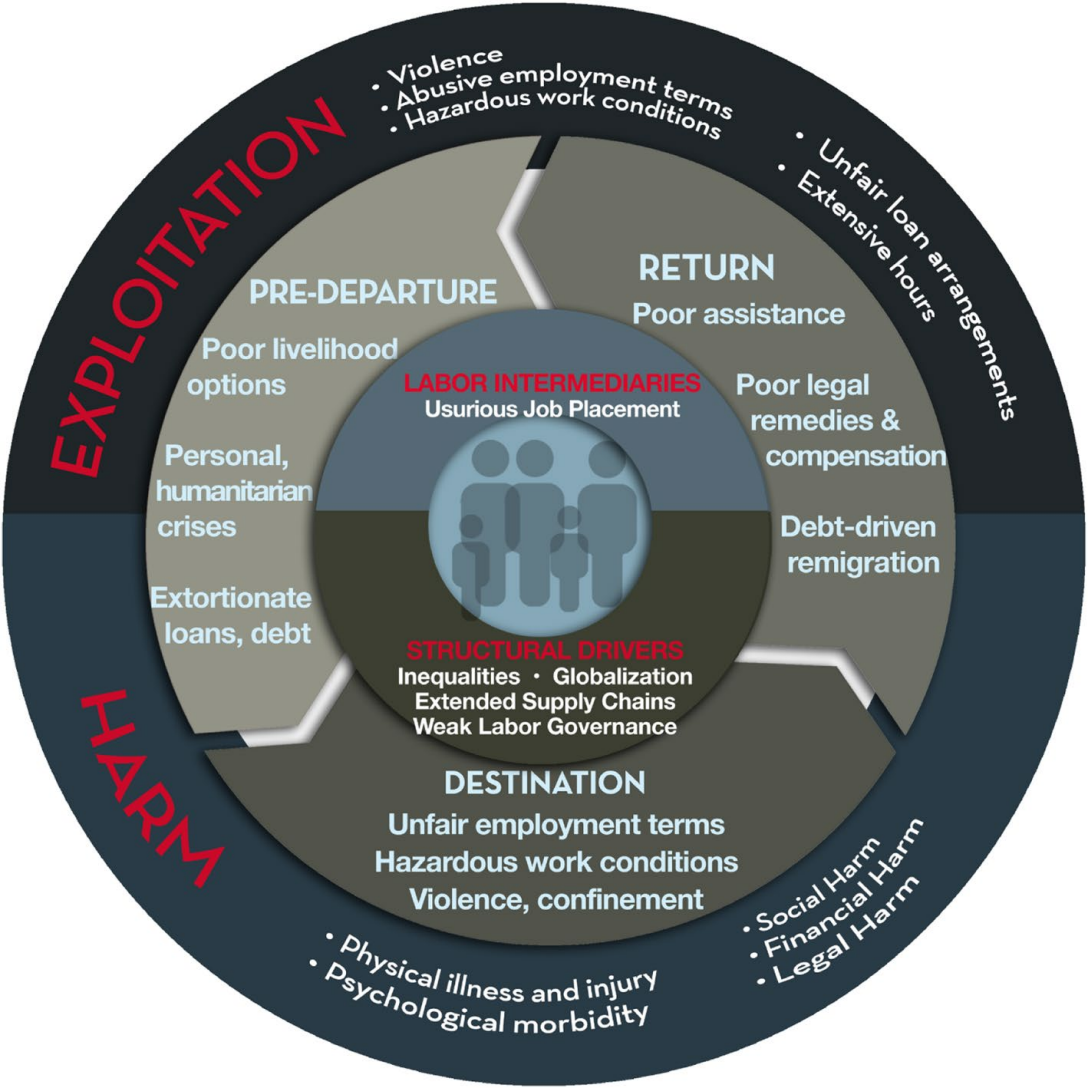
Socioeconomic determinants of labour exploitation and harm. Source: Zimmerman & Kiss, 2017

We used the conceptual model of socioeconomic determinants of labour exploitation and harm (Fig. 1) [9] to guide the qualitative data analysis.

This model describes the trafficking process as a three-stage pathway, in which risks are exacerbated by individual and contextual factors. The complex, cumulative nature of exploitation is depicted in its association with harm through the migration cycle. Common characteristics of each stage are described in the model. At pre-departure, lack of local livelihood options, politically unstable environments, and groups that intermediate migration influence decisions. At destination, violence, unfair employment terms, poor working conditions, extensive hours and isolation are common in the exploitation of trafficked migrants. In the aftermath of trafficking, insufficient assistance options, poor remedies and lack of compensation sometimes lead survivors to re-migrating in similar circumstances of debt and vulnerability to exploitation [26].

We identified themes that emerged from the interviews about each of those stages: pre-departure, transit, destination and exit/return. The qualitative analysis was conducted using the software QDA Miner Lite. Statistical analysis was performed in Stata/SE 14.2.

## Results

### Pre-departure circumstances: poverty, conflict, neglect and violence

The vast majority of the Nigerian sample with complete data (84% of 87 girls and young women) in the IOM routine dataset reported that they entered trafficking between the ages of 18 and 23. Among Ugandans, almost half of the 76 adolescents (for whom this data was available) entered the trafficking situation as young children (between 0 and 13 years-old). The reported age ranges at the time of Ugandan women and girls’ entry into the trafficking process were 0-8 (33%), 9-11 (13%), 12-14 (14%), 15-17 (3%), 18-20 (3%), 21-23 (13%).

In the qualitative research, all Nigerian and Ugandan adolescents interviewed by our team reported adverse pre-departure circumstances. They described various traumatic events in their family lives, including loss of one or both parents, emotional abuse, physical and sexual violence by caregivers, neglect, sexual exploitation, child labour, witnessing violence, parental alcohol abuse, parental divorce, extreme poverty, hunger and racial discrimination.

In addition to these experiences, Nigerian adolescents also described how the protracted conflict in the Northern states of the country affected their lives. Six out of the eight interviewees reported being forcibly displaced by Boko Haram. Three participants had one of their parents killed by the militia. One of the adolescents was kidnapped and subjected to forced marriage with one of the group’s combatants.

*…when Boko Haram attacked us and killed my mother, they also take my brothers away, we ran to escape the attack. We arrived in* (place) *where the military allowed us to stay* (Nigerian adolescent, trafficked nationally for sex work at age 11).

*…my dad was killed by Boko Haram, after the Boko Haran took us inside the bush, the military helped us escape and we came to [the refugee] camp then my mother also died* (Nigerian adolescent, trafficked nationally for forced marriage at age 14).

The Ugandan adolescents did not mention conflict, but some described pervasive community-based violence and unrest.

*Like theft, murder, alcoholism and getting other drugs* (Ugandan adolescent, trafficked nationally for domestic work at age 16).

None of the adolescents interviewed finished secondary school. Most left school before finishing their primary studies, and a few never attended formal education. The majority attributed the interruption of their studies to their family’s lack of money to pay education fees. School discontinuation happened in a context of poverty, family breakup and child neglect.

*I would be in school and then I would have no school fees so I would drop out and then I would get the money when the students were about to do exams so I got fed up and dropped out* (Ugandan adolescent, trafficked nationally for domestic work at age 17).

*He paid my school fees but when he realised that he had no* [money for] *school fees because he had so many children of his own, he told me that ‘stop schooling so that I can pay school fees for my own children’. So I stopped schooling and up to this day I have not gone to school* (Ugandan adolescent, trafficked nationally for domestic work at age 13).

*Actually, I love my mum more than my dad because she is the only one paying my school fees. When I was in JSS 2 going to JSS 3, I asked my dad about my school fees and he said he does not have any money. My father really hates me, since when they gave birth to me, he does not like me. As in he does not want to see me, he does not want to be talking to me, he just hates me. It’s just my mum, she really tried and struggled until I got into SS 2 and when I ask, he will be treating me as if I’m not his biological daughter* (Nigerian adolescent, trafficked internationally for sex work at age 17).

In Nigeria, six of the eight adolescents interviewed reported that one or both of their parents died. In Uganda, four girls had lost at least one parent. The other four Ugandans interviewed reported that their mothers abandoned or neglected them.

*I was staying with my sister, at first I was staying with my parents and parents died of accident, they both died on the same day and I started staying with my sister* (Ugandan adolescent, trafficked nationally at age 16, did not reach work destination).

*When they separated I do not know where my mother went to because for about one year I did not know where my mother was. I also did not know where my father was* (Ugandan adolescent, trafficked nationally for domestic work at age 17).

*I asked her about my age, because my mother was a drunkard…at home we were living in very poor conditions, we did not have anything, no form of assistance or source of livelihood (…) she was a drunkard and there reached a time when she abandoned us at home…she left us, went and got married somewhere else* (Ugandan adolescent, trafficked nationally for domestic work at age 15, did not reach work destination).

*Yes, when I was eight years old my young brother was five years old, my mother left and we remained with my father but my father did not have a regular job, he would only take alcohol but he had a huge chunk of land. At times he would tell us to dig or to harvest bananas.* (Rwandan adolescent, trafficked internationally for domestic work at age 15).

Emotional, physical and sexual violence were common pre-departure experiences among Nigerian and Ugandan interviewees. The perpetrators were close family and extended family members.

*They would not give me food, they would harass me and even beat me, and they were not treating me like a child to their sibling* (Ugandan adolescent, trafficked nationally for domestic work at age 13).

*She would mistreat me a lot and hurl insults at me so I got fed up and ran away and I went to my aunt’s place in* [another town]. *I was living with my aunt I felt that I was a burden to her so I found somewhere to work* (Ugandan adolescent, trafficked nationally for domestic work at age 17).

Repeated experiences of abuse and neglect often led to the perception that they were not loved or wanted by close family members.

*One day I asked my mum if he’s my father and she said yes, why I’m I talking like that. I said the way the man is treating me; it’s not like he’s my real father. (…) But my step-mum hates me, anything that I do in the house, she will just be shouting on me and me insulting me. Since when I left that place, when I was in Italy she was talking to me with love because they were looking at me to sell my body and send them money, that’s what they want me to do but I told them that is not my life, that’s not the life that God chose for me and they started insulting me* (Nigerian adolescent, trafficked internationally for sex work at age 14).

*The reason why I did not want to go my mother is that my father is dead and there are quarrels and I am a twin, my twin brother is fat, short and light skinned whereas I am small in size and tall so my father’s relatives said that I do not belong to them, that they do not have children like me in their family and so they told my mother that she should abandon me if she wants peace with them, I was very small and so my mother took me to her mother, I grew up thinking that my grandmother was my mother (…) That is the problem I experienced, I used to wonder that why are they discriminating against me, why do they say that I am a mulalu* (in the original meaning crazy, mad or insane) *… (*Rwandan adolescent, trafficked internationally for forced marriage at age 16).

In most cases, the challenging circumstances in which many were living and the feeling of rejection influenced their decision to migrate.

*Maybe about step mother, what hurt me most at the time when I was living with her is this: my father loved us as children but that woman would tell us that your father whom you are clinging on to is not going to love you, he will love you today and tomorrow he will be minding about my children. And that would hurt me. She would even say that your mother is a witch and she would spread those lies in the village and yet they were not true; that is what prompted me most to leave home (*Ugandan adolescent, trafficked nationally for domestic work at age 17*).*

Almost all interviewees reported that a relative, family friend or neighbour arranged their migration. Half of the Nigerian interviewees reported that relatives were involved in arranging their migration, whereas two referred to a friend of their mothers. In a few Nigerian cases, the parents of the adolescent were actively involved in arranging and sometimes coercing migration.

*When they came, they asked my father that they need a girl they can take to outside country. They were now disturbing me because of that. My dad and my mum were now quarrelling me because of that, that I don’t want to go and change our lives. They were now forcing me that I should make our lives better and disturbing me, because of that I could not concentrate on my studies. I told them that that’s not my work and that’s not my dream. I said I want to be a footballer. They said (…) what I’m I doing in this place, I’m here and wasting their money. I obeyed them as my parents* (Nigerian adolescent, trafficked internationally for sex work at age 17).

*It was one week later when I got there, she told me the kind of work I was doing* [sex work] *which I disagreed with her. So she called my mom and she told my mum that I was not ready to do anything. So when I spoke to my mum I never knew that she was involved, she told me that I should try to do it which when I heard, the thing really broke my heart (*Nigerian adolescent, trafficked internationally for sex work at age 14*)*.

In Uganda, two described that the intermediaries were relatives. In three cases, they were neighbours. In another two cases they were acquaintances or family friends and, in one, the recruiter was a stranger.

*We persevered and stayed with him…that is when this woman [the recruiter] came and told me… she told my step father, why don’t you give me that child and I take her to Kampala, to work…He asked me whether I want to go to Kampala to work…and I told him that, Yes I want to go and work… I bid farewell to my little sisters and told them that once I earn a lot of money, I will send them some money that will sustain them…* (Ugandan adolescent, trafficked nationally for domestic work at age 15, did not reach work destination).

### Transit conditions: sexual violence, fear and hunger

Transit circumstances were particularly difficult for Nigerians, both for those migrating internationally and internally. Five girls reported sexual violence during transit and three mentioned hunger. Adolescents escaping conflict-related violence engaged in long walks in the bushes for up to five days.

*And the girl we look like twins but we are not twins. After they catch us, the police, they started touching their body. So lucky they took the girl to another place and took me to another place. It’s only me and three of us that they took to the place that they are going to while the second girl the police took her to their own house. When they reached there, they said that they sleep with them… I now said I thank God for my life, I would have been among the girls they could have raped. Some of the girls that they raped, she was now vomiting, they now said she’s pregnant. I was now happy for my life. I now said that if I know this is what I’m going to pass through, I would not have come to this place at all. And they was beating people, as in they was killing people as if the person was cow. Many things was happening, and if somebody falls from the car, they will leave the person until the person will just die in the desert* (Nigerian adolescent, trafficked internationally for sex work at age 17).

*The trip was difficult, we were stopped along the road by armed men because we do not have ID cards and they ask us to sleep with them* (Nigerian adolescent, trafficked nationally for sex work at age 11).

*The trip started the night the Boko Haram attacked [our village]. They burn down our village, we ran and walk in the bush for five days before we reach [the city]* (…) *they raped me in the bush because I don’t have food* (Nigerian adolescent, trafficked nationally for sex work at age 11).

A few Ugandan adolescents also reported dangerous and uncertain transit conditions, where sexual violence and hunger were common experiences.

*I left* [the village] *late and reached Kampala at around midnight but there was nowhere I could go. The money was even over. The money that I used from* [the village] *up to here was over and a certain bodaboda* [riders of motorbike taxis] *… I was now just walking around. He asked me that where are you going and I told him that you leave me alone, he told me that you are lost you come and I take you to the police station, I thought that the bodaboda was taking me to the police station yet he was taking me to his home, and he locked me inside his house and came back to work in town, he came back late at night and he raped me, he chased me away and told me never to return to his place and yet it was my first time to go there, I did not even know that place* (Ugandan adolescent, trafficked nationally at age 16, did not reach work destination).

*I had made 15 years…He told his colleagues that I am mature…one of them told me that he can sleep with me and then let me go…I asked him what he meant…and he told me that he wanted to take my virginity, and I refused and asked them to let me go…they all ganged up on me, and three of them defiled me…I started bleeding and they pushed me out of the shack, one of them kicked me hard and I left behind my clothes and sandals….I walked while bleeding…I sat somewhere on a round concrete…bleeding with no one to help me…my dress was all soiled in blood and I did not have any other dress…I did not have anything to do…that is why I sat down. A woman came and asked me, what is wrong with you, are you in your periods and do not have sanitary pads? I told her while crying that, no I have been defiled by security personnel that are working on the roads…* (Ugandan adolescent, trafficked nationally for domestic work at age 15, did not reach work destination).

Neither the Nigerian nor the Ugandan adolescents had a support network, or even contacts at destination that they could use when facing adversity during transit or at destination.

### Destination: deception, abuse and exploitation

The vast majority of Nigerian girls and young women (for whom IOM data was complete) were trafficked into sex work (91.3%). Approximately one in ten engaged in forced labour. None were trafficked into forced marriage, criminal activities or organ removal. More than half of girls and women were trafficked into Russia (55.2%) and almost one quarter were exploited in Eastern Europe. The vast majority of girls and women (81.8%) who were trafficked for forced labour were exploited in Morocco, with 83.3% also performing sex work.

The majority of Ugandan children and adolescents with available data were trafficked into forced labour, whereas most youth were sexually exploited. Almost a third of children and a tenth of adolescents were involved in criminal activities. Almost all children and adolescents were trafficked within Uganda. All young women were trafficked internationally, mainly to Malaysia and Thailand. Only one case of sexual exploitation within Uganda was identified, involving an adolescent (Table 1).

**Table 1:** Migration destinations and type of exploitation among female children, adolescent and youth by country (frequencies)a

		Nigeria N(%) *N *= 146	Uganda N(%) *N*=
Type of exploitation	Forced labour	11/106 (10.4)	59/71 (83.1)
	Sexual exploitation	96/106 (90.6)	15/75 (20.0)
	Criminal activities	-	12/71 (16.9)
Destination	Denmark	8/99 (8.0)	-
	Ecuador	1/99 (1.0)	-
	Egypt	1/99 (1.0)	-
	France	6/99 (6.0)	-
	Iraq	-	1/93 (1.1)
	Ireland	1/99 (1.0)	-
	Italy	8/99 (8.0)	-
	Kenya	-	2/93 (2.2)
	Kuala Lumpur	-	6/93 (6.5
	Malaysia	12/99 (12.0)	19/93 (20.4)
	Morocco	12/99 (12.0)	-
	Norway	4/99 (4)	-
	Russia	53/99 (54.0)	-
	Spain	2/99 (2)	-
	Thailand	-	6/93 (6.5)
	Turkey	-	1/93 (1.1)
	Uganda	-	58/93 (62.4)
	United Kingdom	1/99 (1.0)	-

^a^ CTDT data

In the qualitative sample, four Nigerian adolescents were trafficked into sex work, two into domestic work, and one was working in a restaurant. The age at which they migrated ranged from 9 to 17 years-old. Three adolescents were trafficked internationally, two into Italy and one into Chad. Three girls were trafficked into Nigerian cities or towns, and two were exploited while living in internally displaced people (IDP) camps.

[Auntie] *took me* [into town from the camp] *and they took us to another part of town every night to meet with men. We stay there till morning and come back to home. At time* [auntie] *will ask me to go and meet men in town and I will stay with him for two days.* [Auntie] *said that is the only job* (Nigerian adolescent, trafficked nationally for sex work at age 11*).*

*I still refused to do it so she chased me outside to go and stay outside, you know the weather is cold so I had to come inside to beg her. She convinced me that other girls are doing it and they are using the money to build house for themselves. So I had to do it for like six months and which she beat me and insult me and all these kind of things. Even when I come back, I will have to do the house chores, I will clean, wash* (Nigerian adolescent, trafficked internationally for sex work at age 14).

*I lived in* [Auntie’s] *tent after we arrived in* [the refugee camp]. *I did not have anybody to help me and there is no food. So I lived with* [Auntie] *and she take me out of the camp (…) we go to meet some men at town in the night.* [Auntie] *always left me with them and they will give her the money. She said she is keeping the money so that we can have something to help us in time of needs.* [Auntie] *took me to a lot of military men and other people* (Nigerian adolescent, trafficked nationally for sex work at age 11).

*…cleaning, mopping, cooking, and washing, always eating white rice with no oil, no pawpaw, even I worked she wasn’t satisfy, she beats me all the times* (Nigerian adolescent, trafficked nationally for domestic work at age 16).

One girl was abducted by Boko Haram and forced to marry a combatant. When this man died, she was forced to marry another militant. During her abduction she was also forced to work in agriculture by the armed group.

*We were told we were slave because we have been working for the military and government. We were beaten everyday. Then one day they married me to* [a combatant]. *We live inside a cave. One day* [this combatant] *went to fight with other Boko Haram. He was killed by the Nigeria military. So then I was married to* [another combatant]. *(…) We work every time. In the morning we cook if there is food, we fetch water and we go to farm. (…) We were beaten and they called us unbelievers and that we must marry a believer before we can be saved* (Nigerian adolescent, trafficked nationally for forced marriage at age 14).

Six Ugandans were trafficked internally. Two Rwandans were trafficked into Uganda. Their age at migration ranged was 12 and 17 years-old. Kampala was the main final destination for these adolescents. Seven of the eight girls were trafficked into domestic and care work. One was trafficked for forced marriage and sexual exploitation.

*When I was growing up there was some work that I did not like to do in my life, like doing domestic work or vending cooked food by the road side in the evening. I used to think that a house maid is despised a lot and I did not like that; that is the reason why even when I was growing up I did not want to work as a house maid. Even when my aunt got this job for me I felt a lot of difficulty but I had to do it because there were things that I needed (*Ugandan adolescent, trafficked nationally for domestic work at age 17*).*

*“House girl”* [domestic worker] *taking care of a child, that child was very young…I was supposed to take care of this child when his/her mother goes to work…this was a child…but I was told s/he was a very good child…I was supposed to feed, bath and care for the baby…and put the child to sleep* (Ugandan adolescent, trafficked nationally for domestic work at age 15, did not reach work destination).

*He told us that he had a hotel in Uganda, that he had friends who had hotels in Uganda and that he was bringing us here to work for them and earn money so I said to myself that he has saved me, I was already tired of this place. When we got here he mentioned marriage and I said ‘oh my God’. I thought I was going to work but marriage?* (Rwandan adolescent, trafficked internationally for forced marriage at age 16).

Half of the girls interviewed in Nigeria and half in Uganda were either deceived into migration or about the nature of the work they were expected to perform.

*I did not even know whether it was prostitution. Actually my mum and dad they know but they don’t want to tell me because they know that if I know I can’t even go there, I rather die, that’s why they did not even let me to know that that’s the work they are doing* (Nigerian adolescent, trafficked internationally for sex work at age 14).

*I said I’m seventeen, she* [recruiter] *now said ah that your mummy she’s a very wicked woman. When she said that she’s a very wicked woman, I now said why did you say that? She said do you know the work you are going to do there? Because its prostitution. I said really? say the truth, she said it’s because I love* [you] *that’s why I’m telling you* ( Nigerian adolescent, trafficked internationally for sex work at age 17).

They were also often deceived about the workload, working hours and wages.

*I asked her that will you give me money and she told me that I will give you twenty thousand shillings. I would work for her but whenever she came back from work she would beat me and abuse me. Whenever I would ask her that where is the money that I am working for? She would tell me that I sent the money to your home.* (Ugandan adolescent, trafficked nationally for domestic work at age 13).

*When I went to live with my father’s relative in Chad, I worked in my aunt’s restaurant. We work form morning till late in the night. They do not give us food and money.* (Nigerian adolescent, trafficked internationally for work in restaurant at age 9)

The IOM quantitative data confirms the high prevalence of violence across trafficking cases in both countries (Table 2). Nigerian adolescents experienced more violence compared to children and young women. The main perpetrator of all types of violence in Nigeria was the exploiter. The recruiter was the second most reported perpetrator of physical and emotional violence, whereas sex work clients were the third most common perpetrators of sexual violence. Threats against the women and girls were mainly perpetrated by the exploiter, followed by the receiver. Threats against their family were mostly made by the exploiter and the recruiter. Recruiters were the agents most likely to deceive girls and women. The main perpetrator of all types of violence against trafficked Ugandans was the exploiter, followed by the recruiter. Both Nigerian and Ugandan children, adolescents and women who were sexually exploited also reported having been prevented from refusing clients, refusing sexual acts and denied using condoms.

Working conditions in the trafficking cases recorded IOM and their partners were also abusive, with high levels of reports of violence, threats, restricted freedom, excessive working hours and withheld wages.

**Table 2:** Frequencies of means of control during trafficking among survivors who reported at least one experience^a^

	Nigeria *N *= 31	Uganda *N *= 54
Trafficking experiences	N(%)	N(%)
Physical violence	17 (54.8)	23 (42.6)
Sexual Violence	14 (45.2)	4 (7.4)
Emotional violence	-	20 (37.0)
Threats	14 (45.2)	15 (27.8)
Threats to family	10 (32.3)	2 (3.7)
Deception	23 (74.2)	33 (61.1)
Debt bondage	16 (51.6)	7 (13.0)
Wages withheld	13 (41.9)	15 (27.8)
Restricted freedom	22 (70.8)	4 (7.4)
Restricted access to food	11 (35.5)	4 (7.4)
Drugs	3 (9.7)	1 (1.9)
Alcohol	2 (6.5)	3 (5.6)

^a^ CTDT data

In the qualitative interviews, most Nigerian interviewees reported high levels of violence during trafficking. The two adolescents who were exploited in sex work in Italy reported threats, emotional abuse and physical violence by the “madams” or pimps. The two girls who were sexually exploited in Nigeria reported being raped. The adolescent who was abducted by Boko Haram reported physical and sexual violence by her captors.

*I was not treated properly, I was raped and they send my money to my uncle who lives in [town]* (Nigerian adolescent, trafficked nationally for domestic work at age 16).

*Life in [the camp] was very difficult, Auntie made me sleep with men every time. When I got pregnant she told everyone that I have been sleeping with men. She chased me out of the tent* (Nigerian adolescent, trafficked nationally for sex work at age 11).

*But the other woman, I was into situation where the woman started insulting me because she did not get what she want from that the prostitution, now I told the women that my life is not for prostitution I can’t do it…* (Nigerian adolescent, trafficked internationally for sex work at age 17).

One Ugandan adolescent, trafficked into domestic work, reported sexual violence by members of the household where she was working. Two girls experienced physical violence by employers. All adolescents who reached the work destination reported emotional abuse by employers.

*For instance, a boy trying to rape you…I stayed there, I had my own room but most of them would go for outings and maybe one of them would remain behind or even two of them; but remember that one of them might be upstairs while the other is down, I would sleep unknowingly and then hear someone walking outside, I would open the door only to find that it is one of the boys who had come to disturb me…That is why I did not like such kind of work* (Ugandan adolescent, trafficked nationally for domestic work at age 17).

*She would start abusing me and when she would get to the house and find that something is not in order, she would abuse me (*Ugandan adolescent, trafficked nationally for domestic work at age 13*).*

The Nigerian girls who were sexually exploited also had restrictions imposed in their freedom of movement.

*No, she don’t (sic.) allow me to communicate with anybody, except maybe she wants to greet my mum, then she will collect the phone* (Nigerian adolescent, trafficked internationally for sex work at age 14).

*No she will not allow me to go out, I just eat, watch TV and sleep. In the evening I will dress up and go to work (…) I don’t have a phone, they collect my phone, they say they don’t want me to run* (Nigerian adolescent, trafficked internationally for sex work at age 17).

*…whenever [Auntie and friend] are going out or travelling she will lock me inside the house with only small food and water. I am always hungry and thirsty* (Nigerian adolescent, trafficked nationally for domestic work at age 16).

Only one of the Ugandan adolescents reported restrictions in her freedom.

*No, she would not allow me to move around, I was not even allowed to play* (Ugandan adolescent, trafficked nationally for domestic work at age 13).

Working hours were particularly taxing for the Nigerians trafficked into sex exploitation, with overnight shifts and sometimes domestic work required during the day. In Italy, the adolescents would work in the streets from sunset to sunrise. They were in debt bondage and their wages were taken from them to compensate the trafficker for the costs of their journey.

All the Ugandan adolescents who reached their destination had wages withheld.

Both Nigerian and Ugandan adolescents shared with our team their mental suffering not only during trafficking, but also before they left their homes. They reported feeling hurt, hopeless, sad and lonely. They also mentioned losing their appetite, having suicidal thoughts and attempting suicide.

*One day I even wanted to kill myself because I’m really tired. She was the one that encouraged me that I should not even try it, she encouraged me as* [if she was my] *mother and* [I was] *her blood daughter.* (Nigerian adolescent, trafficked internationally for sex work at age 17)

*I used to cry most when I was washing clothes, the clothes were so many and they were for adults. At times I would stop washing, sit down and start crying but then I would say to myself that even if I cry, I will still wash the clothes. Crying will not benefit me. So I would say Lord you are the potter, then I would sit down and wash but if I had kept on crying I would not have been able to wash them (*Ugandan adolescent, trafficked nationally for domestic work at age 17*).*

*I was badly affected, I even got to the point of committing suicide…I wanted to die, I thought of taking poison…in fact I took the poison, I wanted to die, it is the neighbour that gave me milk and she told me to take it. She counselled me, and told me that this is not the end of the world…life will be better one day…She told me that I am still a young child, the beautiful things are yet to come…I then became strong (*Ugandan adolescent, trafficked nationally for domestic work at age 15, did not reach work destination*).*

### Exit and entry into assistance

Nigerian and Ugandan adolescents exited the trafficking situation under diverse circumstances. The police were involved in three Nigerian cases. One girl managed to escape. Two adolescents were let go because they either had an accident or were pregnant.

*…I just felt that everybody abandoned me, so I have to fight on my own. I later started thinking what can I do to get out of the situation before I came up with an idea to just call the police. So I just thought of calling the police and explaining everything to them so when I call them, they traced they call and they came to where I was.* (Nigerian adolescent, trafficked internationally for sex work at age 14).

*Police came and arrest (sic.) us before the NAPTIP people came to take us (*Nigerian adolescent, trafficked nationally for sex work at age 11*).*

In Uganda, four of the eight adolescents interviewed were able to count on the help of neighbours, acquaintances or strangers. The police referred four Ugandan girls to services which offer assistance to trafficking survivors.

*There was a woman who used to sell milk, she once asked me that would you like to go back to school and I told her that if I get the opportunity I would love to go back. Then she told me that these people are going to teach tailoring, hair dressing and such skills. (…). One day my boss had rebuked me and when she rebuked me I became so angry and remembered what I had been told so I did not tell her anything. The next time that I saw the milk woman I told her that I will come and then she organised everything for me… (*Ugandan adolescent, trafficked nationally for domestic work at age 17*)*.

*One day she came back home and beat me up severely and then I told her that ‘sijja kukigumikiriza’ [I will not tolerate this] so I went to the police station and told them about it, the police then told me that we are going to take you somewhere. They asked me that what would you prefer taking you back home or taking you somewhere to learn hair dressing. So I told them that I want to learn hair dressing and that is how they brought me up to this place (*Ugandan adolescent, trafficked nationally for domestic work at age 15, did not reach work destination*).*

As the quotes above reveal, both Ugandan and Nigerian participants often had their decision to leave prompted by episodes of violence by their employer or traffickers. Only one Rwandan adolescent referred to the prosecution of her trafficker.

## Discussion

This paper aimed to address the evidence gap on the intersections between violence and exploitation in the migration pathways of Nigerian and Ugandan adolescents and young women to inform policy and interventions. To our knowledge, this is the first mixed-methods study to address this topic in Sub-Saharan Africa.

Our findings reveal the pervasiveness of childhood violence and neglect in adolescents’ lives both before and during migration. Quantitative data collected by the IOM confirms the extent of violence and abuse experienced by girls and young women during migration. Our findings are in line with findings from previous studies in Africa, which detected high levels of violence against women at different stages of the trafficking cycle [27, 28].

The experiences reported by participants also overlap with most of the factors described in the conceptual model of socioeconomic determinants of labour exploitation and harm. Risks identified in our findings align with those described in each stage of the model. In line with the model, adolescents in our study described poor livelihood options, along with circumstances of personal and humanitarian crisis that often motivated their migration. At destination, they faced unfair employment terms, hazardous work conditions, violence and restricted freedom. Upon return/exit, legal remedies and compensation were mostly unavailable [29].

This Discussion section expands on findings for each of the phases in the trafficking cycle, considering implications for policy responses and interventions.

### Pre-departure: adverse childhood experiences

Adolescents who participated in our qualitative research reported a wide range of adverse childhood experiences (ACE) [30] before migration, including pre-migration exposure to physical violence, sexual abuse, emotional abuse, neglect, household substance abuse, parental separation and food scarcity. Nigerian adolescents also experienced additional stressors related to the protracted conflict in the North of the country. In both countries, the lack of family and social support associated with the adolescents’ circumstances hindered their educational opportunities and hampered their safety nets.

Our findings suggest that interventions to prevent or mitigate the negative impact of ACEs - including child abuse, child neglect and household dysfunction - could contribute to prevent unsafe migration of adolescents. This may be particularly important given findings from previous research indicating that a lack of family support and abusive circumstances at home increase the risks of re-trafficking for children and young adults [31]. In High Income Countries, these types of intervention usually combine parental education, social service referral and social support for families [32]. In contexts such as Nigeria and Uganda, interventions that ensure gender equality in universal access to primary and secondary education are also needed [33, 34].

For many participants, migration was the only way to escape domestic and conflict-related violence and guarantee a livelihood. The interplay between social crisis and intersectional vulnerabilities associated with gender, age and class, and coupled with meagre social support, is known to increase adolescents’ vulnerability to further violence and exploitation during migration [35]. Interventions aiming to promote safer migration through awareness raising and empowerment training are unlikely to succeed in these contexts marked by deep power asymmetries between migrants and the agents of their migration (e.g. family members, intermediaries, officials, employers) [36]. Interventions to prevent trafficking need to reduce opportunities for these agents to rely in these power imbalances to exploit adolescent migrants.

### Transit: rights, protection and assistance

In most cases, adolescents reported that family members and/or close social contacts arranged their migration. These finding are in line with global estimates describing that family members are involved in the recruitment of 41% of child trafficking cases (versus 9% of adults’ cases) [37]. Previous research in Nigeria also suggests that lack of parental support increases trafficking risks for children [19]. Although not formally defined as an ACE, the participation of close family members in arranging trafficking of children and adolescents is likely to contribute to the well-researched deleterious effect of early life adversities on health [38, 39].

In our study, most intermediaries who facilitated in-country transport, accommodation or employment during migration were not previously known to adolescents. Not all adolescents relied in such intermediaries for migrating within their country origin. Of those who did, none reported violence by these agents. Violence during transit was mostly sexual, opportunistic and perpetrated by strangers, combatants or officials. Risks of non-partner sexual violence are high in sub-Saharan Africa, with adjusted regional prevalence estimated at 11.4% for East Africa, where Uganda is located, and 9.1% for West Africa, where Nigeria is [40]. For women who experience sexual violence, access to early and comprehensive care is essential, including prevention of sexually transmitted infections, prevention of pregnancy, counselling and forensic examinations [40, 41]. Primary prevention focusing in potential perpetrators is considered the most effective strategy to reduce levels of sexual violence in the medium and longer term [42].

Conversely, violence and human trafficking by organised militia groups, such as Boko Haram in Nigeria, are differently organised and perpetrated. These systematic crimes often take place in contexts where the groups have some territorial control and are embedded in local networks. They often involve victims’ abduction [43].

Unlike in cases of internal migration, adolescents in our study who engaged in cross-border migration reported pervasive violence in transit by different migration agents (e.g. recruiters, officials, smugglers). Previous reports documented the high levels of violence and exploitation experienced by migrants in transit through Libya [44].

 Building on the scarce evidence for prevention of violence during transit, recent WHO guidelines for Europe highlights the importance of ensuring safe passage for migrants, identifying trafficking cases, providing care and protection to survivors, investigating and prosecuting perpetrators, and strengthening the knowledge base (WHO, 2020). Evidence on the implementation and effectiveness of such efforts could help inform future national and cross-border policies and interventions.

### Destination: identification and re-integration

At destination, violence was often used by employers to coerce, control and exploit adolescent migrants. Emotional, physical, sexual and economic violence were reported by participants. Restricted freedoms and social isolation have likely contributed to their permanence in the trafficking situation. In addition, the invisible nature of domestic work and sex work [45, 46] has probably played a role in the lack of official identification and rescue efforts they experienced.

Even without external targeted help, all participants in our study found a way out of the trafficking situation and into assistance. However, many other girls might never speak about the exploitation, abuse and violence that they experienced, let alone seek help. This is true especially in Sub-Saharan African, where the number of undetected cases are one of the highest in the world (OECD, 2018). As Swartz (2014: 195) [46] wrote about commercial sexual exploitation of children, “these crimes are overlooked and underreported because they often occur at the margins of society and behind closed doors”. Hatton [45] described how both domestic work and sex work remain invisible in the mainstream economy. As invisible work, these types of employment are often ignored or overlooked, socially marginalised, economically devalued and legally unprotected and unregulated. According to the author, this invisibility is created by the intersection between social norms that devalue this type of work, legal systems that do not regulate the work, and sociospatial mechanisms that segregate the work. Trafficked female adolescents are at the extreme end of this invisibility spectrum, as socially isolated and politically disenfranchised migrants working in stigmatised and, sometimes, illegal industries. Perpetrators of violence and exploitation against them are most often not held accountable for the crimes they commit, as evidenced by the low levels of traffickers’ conviction in Sub-Saharan Africa [47].

Gender inequality is a defining feature of the type of feminised, invisible work performed by the adolescents and young women in our study. Research shows that gender and age place adolescents at increased risk of sexual and gender-based violence during migration, and of specific types of human trafficking. Opportunities to design policies that are sensitive to gender and age do exist, but to date have been underexplored [48].

Additionally, in low-resource social contexts, re-integration efforts need to consider that return to pre-departure circumstances may lead to further social isolation, exclusion and violence. Re-integration is a costly and complex process that involves recovery and socioeconomic inclusion of human trafficking survivors [49]. Without target investment in viable livelihood and protection options at home, returnee migrants are likely to encounter the same environments of resource scarcity or reduced opportunities that first made them leave, if they return to their communities of origin. The type of skills training that was valued by our participants can potentially help their re-integration into local labour markets. However, the effectiveness of skills training for re-integration depends on the alignment of participant’s skills with available opportunities in local labour markets, along with the quality of the vocational training design and implementation factors [50].

The toll that traumatic experiences leave on the mental health of adolescents is clear in the narratives of our research participants. The consequences of trauma can be particularly severe and long-lasting for adolescents’ developing brains [51]. Experiences of parental neglect and ACEs can aggravate these consequences, causing long-term physical and psychological illnesses, cognitive distortions, risk behaviours, developmental disruption and increase need for healthcare utilisation [38, 39]. Post-trafficking assistance need to include not only mental health provision, but also target the social aspects that perpetuate the cycle of violence, exploitation and abuse in women’s lives.

### Limitations

This article has several limitations, as is the case with most of the academic literature on this hard-to-reach mobile population. The qualitative and quantitative data in our study are not representative of the general population of trafficked female adolescents in Nigeria and Uganda. The recruitment of survivors for each research components relies in the identification procedures used by IOM globally (for the quantitative data) and by IOM partners locally (for the qualitative data).

The quantitative database is used across all IOM regional and country missions as a standardised case management tool. Cases in this database represent the population of survivors identified by IOM and their partners globally, whereas the qualitative data is a purposive sample of cases assisted by local organisations. Identification and referral procedures across and within countries affect the constitution of the sample frame. Because the identification of trafficking survivors is often associated with the visibility of trafficking cases for law enforcement, survivors of sex trafficking (versus, for example, agriculture or domestic work) may be overrepresented in the IOM dataset. This trend is documented, for example, by Jones & Edwards (2017) [52], who call attention to the disproportionate focus of “raid and rescue” operations on women and children in the sex industry. Additionally, because of the routine nature of the IOM data, records for survivors are often incomplete. In spite of these limitations, this data is unique in describing the experiences of a considerable number of female migrants who suffered severe exploitation at the hands of recruiters, traffickers and abusive employers. Caution should also be used in interpreting findings from the qualitative research. We selected a purposive sample of adolescents who were identified as human trafficking survivors and were receiving assistance in post-trafficking services in each country. Internal versus international trafficking is likely to be overrepresented in the qualitative data. Immediate assistance to survivors of international trafficking (e.g. shelters, safe houses) tend to take place largely in the country of exploitation, as the victims’ official identification process may be lengthy. Furthermore, survivors’ collaboration in the prosecution of perpetrators require their permanence in the country, with some survivors resettling in the country of exploitation.

## Conclusions

This study contributes to the scarce evidence on trafficking of young women and girls in Sub-Saharan Africa. Our findings suggest that violence is pervasive not only at destination, but also before migration and during transit. Adolescents low social capital and safety nets increases risk of exploitation, violence and abuse during migration. Labouring in invisible and disenfranchised labour sectors, many will never be identified as trafficked and receive the assistance they need. Trafficking is a complex social phenomenon that requires comprehensive multi-level strategies in prevention and responses. Investment in social protection mechanisms and gender equality can help prevent trafficking of girls and young women in Nigeria and Uganda. However, focussing exclusively in pre-departure strategies is unlikely to stop exploitation and abuse during transit and at destination. Effective prevention will also need to tackle the systemic conditions that makes trafficking of female adolescents invisible, profitable and inconsequential for perpetrators.

## Data Availability

The International Organisation for Migration (IOM) shared with our team the dataset, with non-identifiable observation on a selected number of variables. Since our analysis, however, the IOM made the full dataset available through the Collaborative Data Hub on Human Trafficking (CTDC). These data is available at the CTDC website.

## Declarations

All participants signed an informed consent sheet, following our ethics protocol described in more details in the manuscript. All research procedures were performed in accordance with the Declaration of Helsinki.
